# Physical Activity and Incident Hypertension Among Blacks: No Relationship?

**Published:** 2006-06-15

**Authors:** Dustin T Duncan, Rakale Collins Quarells, Rebecca Din-Dzietham, Cassandra Arroyo, Sharon K Davis

**Affiliations:** Department of Psychology, Public Health Sciences Institute, Morehouse College, and Social Epidemiology Research Center, Morehouse School of Medicine, Atlanta, Ga. Mr Duncan is now with the Department of Society, Human Development and Health, Harvard School of Public Health, and the Center for Community-Based Research, Dana-Farber Cancer Institute, Boston, Mass; Social Epidemiology Research Center, Morehouse School of Medicine, Atlanta, Ga; Social Epidemiology Research Center, Morehouse School of Medicine, Atlanta, Ga; Social Epidemiology Research Center, Morehouse School of Medicine, Atlanta, Ga; Social Epidemiology Research Center, Morehouse School of Medicine, Atlanta, Ga

## To the Editor:

Understanding the role (particularly the mechanisms of action) that physical activity plays in the development of hypertension among blacks is important for risk reduction efforts and public policy actions, especially because blacks bear the highest burden of hypertension, and cross-sectional studies suggest that physical activity may be associated with reduced hypertension in blacks (1). We therefore sought to ascertain this relationship between physical activity and incident hypertension among blacks with a study design permitting causal inference.

We performed a qualitative, systematic review of cohort studies examining the relationship between physical activity and incident hypertension among blacks. We searched for articles in Medline from January 1966 to February 2005, examined all potentially relevant articles, and reviewed the reference lists of those articles. Fourteen studies assessed physical activity and incident hypertension in all populations. Most studies were conducted among whites and were conducted in the United States; four studies were conducted in countries other than the United States and did not include blacks, and four other studies included blacks but one did not report the estimate of the physical activity–hypertension association by race. Only three studies met our inclusion criteria.

Overall, cohort studies confirmed the positive effects of physical activity on hypertension among whites. Five studies found that among whites, physical activity reduced hypertension in univariate analyses but not after adjusting for other covariates, such as age, body mass index, and alcohol intake. Univariate analyses of one study found a relationship between physical activity and hypertension among blacks (2); no such relationship was detected among blacks in multivariate analyses of the National Health and Nutrition Examination Survey I Epidemiologic Follow-up Study (2), Coronary Artery Risk Development in Young Adults study (3), and Atherosclerosis Risk in Communities study (4).

However, the three studies have limitations that threaten internal and external validity. One limitation is possible nondifferential misclassification of physical activity levels due to poor physical activity ascertainment (2,4). To illustrate, one study (4) used a questionnaire that was inappropriate for blacks and women (5). Another limitation is the lack of power to examine the physical activity–hypertension association among blacks because of a smaller sample of blacks, which was highlighted when investigators presented stratified analyses of race, sex, and physical activity levels (2). Other limitations that violate internal validity include the following: 1) the unavailability of data on physical activity between baseline and follow-up (2,3) (i.e., physical activity change possibly indicating greater risk than baseline physical activity, particularly in long follow-ups); 2) self-reported bias (i.e., the possible inaccuracy of self-reported physical activity [2,4] and undiagnosed hypertension [2,3]); and 3) interviewer bias (i.e., the possible inaccuracy of physical activity data collected by interviewers) (3). Furthermore, blood pressure was not measured (hypertension was self-reported) at follow-up in one study (2). Self-report and interviewer bias can result in nondifferential misclassification. The limitations hindering external validity are as follows: 1) one study included only blacks and whites aged 45 to 64 years at baseline (4), a sample representative of a limited subgroup of the black population (and hypertension affects blacks at younger ages than whites), and 2) most blacks in one study were from the South (4), which limits the generalizability of its findings. Given these limitations and the limited number of studies on blacks, we question whether there is no relationship between physical activity and incident hypertension in blacks based on cohort studies.

We would conclude from the published evidence that physically active blacks are not at a reduced risk for hypertension, but we recognize the numerous limitations of the research. Cohort studies did, however, report a biologically plausible inverse association of physical activity with hypertension risk. Widening the criteria of our review to include evidence from cross-sectional studies and controlled clinical trials (6) suggests that there is probably a relationship. For example, controlled clinical trials demonstrate that physical activity is associated with blood pressure reduction among blacks (6). Moreover, a cohort study that assessed blood pressure continuously among blacks (7) suggests that loss of information and power due to categorization may be a major contributor to the lack of association observed in cohort studies with categorical outcomes. All of these limitations may explain the lack of association between physical activity and incident hypertension among blacks.

Additional cohort studies and controlled clinical trials are needed to examine physical activity and incident hypertension among blacks. Longitudinal mechanistic research designs that include the mediating variable of physical activity to hypertension are essential for researchers to develop effective hypertension risk reduction interventions and for policymakers to implement informed and effective policies. This may put us closer to reaching the aims of *Healthy People 2010 —* reducing the proportion of adults with hypertension and eliminating health disparities overall. 
